# Monoclonal anti-CD38 therapy in human myeloma: retrospects and prospects

**DOI:** 10.3389/fimmu.2025.1519300

**Published:** 2025-02-12

**Authors:** Alberto L. Horenstein, Angelo C. Faini, Fabio Morandi, Erika Ortolan, Paola Storti, Nicola Giuliani, Paul G. Richardson, Fabio Malavasi

**Affiliations:** ^1^ Lab of Immunogenetics, Department of Medical Sciences, University of Torino, Torino, Italy; ^2^ Immunogenetics and Transplant Biology, University Hospital “Città della Salute e della Scienza di Torino”, Torino, Italy; ^3^ UOSD Laboratorio di Terapie Cellulari, IRCCS Istituto Giannina Gaslini, Genova, Italy; ^4^ Department of Medicine and Surgery, University of Parma, Parma, Italy; ^5^ Department of Medicine and Surgery, University of Parma & Multiple Myeloma Program, AOU, Parma, Italy; ^6^ Jerome Lipper Multiple Myeloma Center, Dana-Farber Cancer Institute, Boston, MA, United States; ^7^ Fondazione Ricerca Molinette Ets, Torino, Italy

**Keywords:** antibody therapies, CD38 monoclonal antibodies, IgG Fc receptors, ectoenzymatic activity, multiple myeloma

## Abstract

Monoclonal antibody therapy using CD38 as a target remains central to managing human multiple myeloma (MM). CD38 was selected early on as a target for mAb-mediated therapy for MM, driven by findings from an early Cluster of Differentiation (CD) Workshop. The first CD38-targeting antibody to be approved yielded strong trial results, significantly improving survival rates and earning widespread patient acceptance. However, resistance to the therapy later emerged, complicating treatment management. Despite CD38’s still central role in MM therapy, too little attention has been paid to its broader roles–not only as a myeloma marker but also as an enzyme and adhesion molecule in physiology. This review, a collaborative effort between basic scientists and clinical experts, explores some of the lesser-known mechanisms of antibody action and interactions with CD38 at key stages of treatment. The review also highlights the relevance of the MM environment, focusing on the importance of the bone marrow (BM) niche. The goal is to identify new agents whose unique properties may enhance tumor eradication. By gaining a deeper understanding of interactions between therapeutic antibodies, myeloma cells, and the tumor microenvironment (TME), it is hoped that previously unrecognized vulnerabilities within the disease may be revealed, paving the way to more effective treatment strategies.

## 1 Introduction

*In vivo* therapy based on monoclonal antibodies (mAbs) represents the natural extension of their widespread use *in vitro*. The unique specificity of mAbs is complemented by their adaptability to industrial production, which forms the basis for an optimal biopharmaceutical.

The selection of CD38 as a target molecule was a key step in the design of a mAb-mediated therapy for human multiple myeloma (MM). This choice was primarily influenced by the main characteristics of CD38, which were initially identified at an early Cluster of Differentiation (CD) Workshop as a T-cell activation antigen. Subsequent research confirmed its high expression in MM cells. Additionally, the molecule is particularly well conserved within the Caucasoid population, exhibiting limited genetic polymorphisms.

CD38 is expressed by the majority of MM patients and is generally maintained during the *in vivo* development of the disease (1). Mainly in virtue of these characteristics, CD38 has been adopted by pharmaceutical companies as an ideal target for antibody-mediated *in vivo* therapy of MM, a diagnosis that is becoming increasingly common worldwide. Since the inception of the project, attention has predominantly focused on CD38 as a marker, while its roles as an enzyme and an adhesion molecule have received more limited consideration (*vide infra*). The first therapeutic mAb, Daratumumab–a fully human immunoglobulin (IgG)–delivered strong trial results and was rapidly approved by the American FDA for *in vivo* use, with Janssen as the drug provider. The results obtained confirmed the effectiveness of CD38 as a target, showing a marked improvement in survival rates (2). The enthusiasm derived from the success of clinical applications was matched by its warm acceptance by patients, as side effects were relatively mild. However, early cases of resistance to the antibody therapy began to emerge, posing problems for treatment management.

This review aims to describe some of the lesser-known mechanisms of action of the antibodies and their interactions with CD38 at key stages of treatment. Furthermore, we will re-examine the enzymatic functions of CD38 in the context of the immune response. Additionally, we will address aspects initially not considered in detail, such as the role of different families of IgG Fc receptors (FcRs), which may influence interactions among mAbs, the environment, and MM cells.

Further structural analysis of the therapeutic antibodies will also be conducted to identify potential areas for improving their efficacy. Currently, the number of approved therapeutic reagents is significantly growing in the last years with Daratumumab and Isatuximab (from Sanofi) leading the way. Felzartamab (from I-Mab), already approved for treating membranous nephropathy, is undergoing trials for additional diseases. For more details, see reference (3).

The final section will focus on the MM environment, with particular attention to the bone marrow (BM) niche, aiming to identify new players and leverage their unique properties to enhance tumor eradication.

The medical community widely expects that deeper insights into the interplay between therapeutic antibodies, myeloma cells, and the tumor microenvironment (TME) will uncover previously unrecognized vulnerabilities of the disease, opening new avenues for treatment.

## 2 Available evidence

### 2.1 The CD38 molecule

CD38 is almost ubiquitously expressed, found at varying levels across most tissues, and present both on the cell surface and within cytoplasmic compartments. The characterization of CD38 has been an extensive process, revealing its high expression on the plasma membranes of both normal cells and malignant myeloma cells. Although the tissue distribution of CD38 has been widely studied and described, its functions remain only partially understood. Initially discovered in connection with the T cell receptor (TCR), early research was shaped by this association (4). CD38 was first classified as a signaling molecule, with its cell activation capability suggesting the existence of a natural ligand, later identified as CD31 (and also hyaluronic acid) (1).

Further analysis of the CD38 sequence revealed a remarkable homology in amino acid structure with a cytoplasmic adenosine diphosphate (ADP)-ribosyl cyclase enzyme previously purified from *Aplysia* (4), suggesting a potential similar function in mammals. This hypothesis was confirmed when human CD38 was found to act as an enzyme with NAD^+^-glycohydrolase activity, consuming nicotinamide adenine dinucleotide (NAD^+^) and producing ADP ribose (ADPR), cyclic ADPR (cADPR) and nicotinamide (NAM) under neutral conditions, and nicotinic acid adenine dinucleotide phosphate (NAADP^+^) at low pH when consuming phosphorylated NAD^+^ (NADP^+^) (4, 5) (v*ide infra*).

#### 2.1.1 Cell surface expression of human CD38

**Figure 1:**
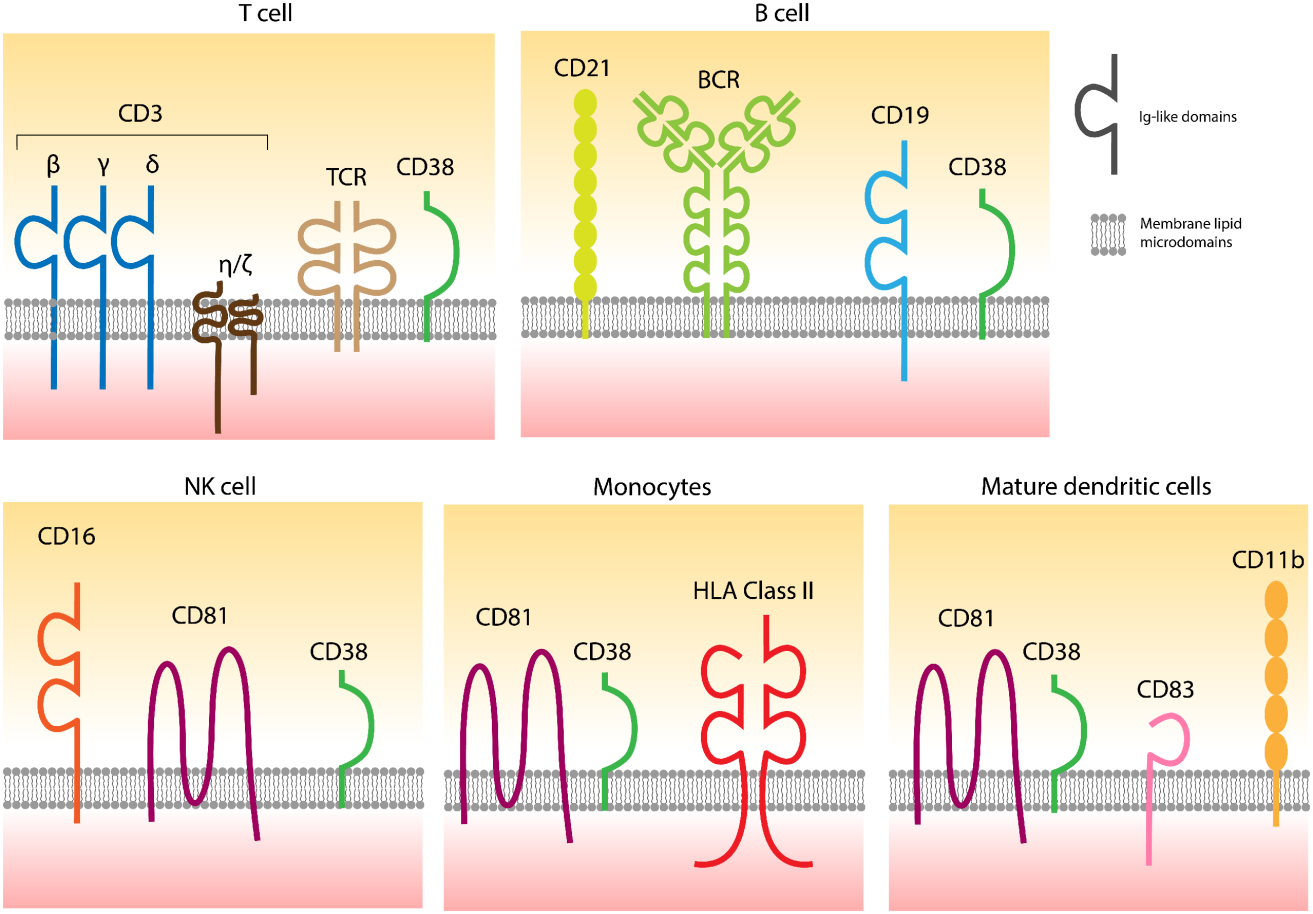
CD38 is localized in specific domains of the cell surfaces containing different receptors, also varying according to the lineage. The links between CD38 and distinct receptors are confirmed, while functional connections are partially hypothesized. Adapted from (1).

Extracellular CD38 is a type 2 transmembrane molecule, approximately 46 kDa in size, featuring a short cytoplasmic tail that grants high mobility within the liquid bilayer, facilitating the formation of soluble forms of CD38 (6). This flexible anchorage is complemented by selective localization in specific domains of the cell membrane, which varies according to lineage (**Figure 1**). The properties of membrane-bound CD38 and its interactions with different surface receptors are summarized in reference (1).

Cell surface expression of CD38 can fluctuate in response to physiological events, such as cellular activation or pharmacological interventions. The interaction between therapeutic antibodies and CD38 on neoplastic cells is inherently dependent on the availability of molecular targets. Consequently, early strategies to enhance the efficacy of MM therapies focused on increasing the number of CD38 targets available for antibody binding.

Various approaches and modalities have been employed to regulate CD38 surface expression on target cells.

##### 2.1.1.1 All-*trans* retinoic acid (ATRA)

CD38 expression can be modulated both *in vitro* and *in vivo* through the nuclear retinoic acid receptor α (7). ATRA, a vitamin A derivative approved for *in vivo* use, has proven effective in enhancing CD38 levels in several *ex vivo* myeloma cell preparations, leading to improved efficacy of therapeutic antibodies (8). However, its *in vivo* efficacy in combination with Daratumumab was called into question in a clinical trial involving relapsed/refractory (RR) MM patients. In this trial, CD38 re-expression was too transient to significantly impact clinical outcomes, at least in the patient cohort studied (9).

##### 2.1.1.2 Histone deacetylases (HDAC)

Another compound investigated for its capacity to regulate CD38 expression is the HDAC inhibitor Panobinostat. Unlike ATRA, Panobinostat upregulates CD38 expression on myeloma and on lymphoma cells, while sparing normal cells. When combined with Daratumumab, Panobinostat boosts antibody-dependent cellular cytotoxicity (ADCC) in both cell lines and primary MM cells. In therapy, Panobinostat induces CD38 upregulation, thereby improving the anti-myeloma effects of Daratumumab (10). Similarly, Ricolinostat, another HDAC inhibitor, increases CD38 expression on MM cells without affecting normal cells. However, Ricolinostat does not affect myeloid-derived suppressor cells (MDSCs), which are depleted by this inhibitor (11).

##### 2.1.1.3 JAK-STAT signaling pathway

The JAK-STAT pathway can influence CD38 regulation through its signaling activities. The *CD38* gene features an atypical promoter responsive to retinoids, vitamin D, and various cytokines in circulating cells. In MM, IL-6 activates the JAK-STAT pathway. JAK-STAT3 signaling down-regulates CD38 expression, whereas JAK-STAT1 signaling promotes its upregulation. Although these effects are less pronounced than those seen with ATRA, STAT3 inhibitors or STAT1 activators are being explored as promising candidates for combination therapies with Daratumumab (12).

##### 2.1.1.4 Degradation of CD38 mRNA

Research involving microRNA (miR) has demonstrated that noncoding RNA can influence CD38 expression, with miR-26a and miR-140-3p being key candidates for investigation. In MM cells, miR-26a is downregulated. Using mimics or antisense nucleotides to inhibit miR-26a can elevate CD38 expression in MM cells with low surface levels of CD38. Conversely, manipulating these miRs can enhance CD38 expression via cell effectors (13).

##### 2.1.1.5 Immunomodulatory imide Drugs (IMiDs)

IMiDs are key components of standard myeloma therapy, especially in combination with mAbs. Their effectiveness is largely attributed to the significant upregulation of surface CD38, achieved by degrading Ikaros and Aiolos. This leads to increased CD38 mRNA transcription, which in turn favors surface CD38 expression and, consequently, improved binding and lytic potential of the antibody (14).

##### 2.1.1.6 Other potential modulators of CD38 surface expression

Surface levels of CD38 rapidly decline following *in vivo* treatment with mAbs, displaying prolonged recovery times that contradict the expected rapid re-synthesis of the molecule or mobilization from internal stores (1). This behavior may also vary according to the normal CD38^+^ effectors.

These effects may be due to the unique localization of CD38 within specific membrane domains of effector cells. Indeed, CD38 has been implicated in the formation of immune synapses in T lymphocytes. Early experiments, later confirmed through various technical approaches, identified two distinct pools of CD38: one on the cell membrane and another within recycling endosomes (15). This distribution pattern is also observed in monocytes (16), mature dendritic cells (15, 17), and NK cells, where CD38 co-localizes with CD16, a key molecule for their activation (18). Comparable effects are also noted with CD28H (19).

Overall, these observations indicate that CD38 influences multiple functional events in NK cells, T lymphocytes, monocytes, and dendritic cells by interacting with various immune receptor systems.

##### 2.1.1.7 Antibody-induced CD38 modulation

CD38 expression during antibody therapy can be studied by examining how the antibody binds the target. The very short cytoplasmic tail of CD38 allows for flexible movement within the cell membrane. At 37°C, therapeutic antibody binding increases the molecule’s ability to float within the membrane and - as known from traditional immunology - the antigen/antibody complex tends to accumulate at one pole of the cell. This polar aggregation is also known as “capping” (20). The consequence is that antibody ligation is followed by the release of microvesicles (MVs) containing membrane rafts with CD38 and the bound antibody, thereby reducing surface levels of CD38 (21). This mechanism may contribute to antibody refractoriness. Polar aggregation is most pronounced when therapeutic antibodies are presented to the target as being bound by a FcR present on normal effector cells. *In vitro* models using polyclonal anti-human IgG mimic the so-called “armed mAb” scenario observed *in vivo*. First, polar aggregation induced by an armed mAb results in a significant re-distribution of CD38 on the membrane and a marked reduction in its expression. Second, molecules accumulating at the pole tend to be extruded through protrusions in the myeloma membrane and eventually released into biological fluids as MVs. These MVs carry not only CD38, but also CD39, CD73 and CD203a ectoenzymes, inhibitory complement receptors CD55 and CD59, the checkpoint inhibitor PD-L1, and the myeloma differentiation marker CD138 (21). The fate of these MVs is determined by specific constraints such as environmental conditions, molecular composition, or cellular interactions. Deriving from cell membranes, they are able to cross vessels and tissues, interacting with monocytes and MDSC (both rich in high affinity FcRs), T lymphocytes, and NK cells, the latter expressing CD16 (low affinity FcR), where they are internalized and release their cargo. These interactions may partially explain the development of antibody refractoriness and resistance.

In addition to reducing surface CD38, antibody-induced modulation may involve two other mechanisms: i) signal transduction that activates intracellular signaling pathways, and ii) cargo transfer. MVs can carry a variety of molecular components, among which coding genes (DNA), non-coding RNAs (ncRNAs), RNAs [including messenger RNA (mRNAs) and microRNAs (miRNAs)], proteins (including enzymes, structural proteins and signaling molecules) nucleotides and other metabolites.

NK cells, in particular, show a marked and sustained decrease in surface CD38 following antibody treatment. Studies with Isatuximab have demonstrated that the cell population appears to be initially activated but is then modulated by the action of a gene set involved in the control of the cell cycle. This was confirmed through molecular analysis of the content of NK cells (22). As a result, NK cells tend to have a modified phenotype and lose surface CD38 while retaining partial cytotoxic activity (23).

### 2.2 The IgG Fc receptors

Another important aspect to explore in detail is the structure of the therapeutic antibody itself. As an IgG, the antibody has two main functional domains. In addition to the domain responsible for binding the specific epitope [*i.e.*, monovalent Fab or bivalent F(ab’)_2_], the second domain (*i.e*., Fc) interacts with cell surface receptors (*i.e*., IgG FcRs) with varying affinities and specificities. The interactions between the Fc region of the therapeutic IgG and FcRs on surrounding effector cells are particularly relevant in the MM environment. Most effector cells express different FcRs, while only the MM cells are FcR-negative.

The signals triggered by the antibody in normal effectors, which are predominantly CD38^+^ and FcR^+^, are one of the most important findings obtained from *in vivo* MM therapy. FcR-mediated functions control a broad array of antibody-driven therapeutic mechanisms, including complement-dependent cytotoxicity (CDC), antibody-dependent cell cytotoxicity (ADCC), antibody-dependent cell phagocytosis (ADCP), and the induction of cell death by apoptosis. These cytotoxic activities have been extensively analyzed in references (2, 24, 25)

Paiva et al. analyzed the effects of Isatuximab, the second anti-CD38 reagent approved by the FDA, on NK cells, which express CD16, a low affinity FcR (22). By using Fc blockers, they demonstrated that NK cell activation is significantly influenced by transmembrane signaling mediated through antibody ligation. The ability of CD38 to transduce signals aligns with early observations of the CD38 molecule (1) and supports the hypothesis that a number of the therapeutic effects, at least in the case of Daratumumab, is reliant on extracellular signaling channeled by CD38. A recent version of Isatuximab with an improved Fc domain, for increased affinity for CD16 and CD32, has recently been tested (26).

Overall, these results suggest that some of the therapeutic actions of the antibody are mediated by direct signaling through the CD38 molecule, without the effects driven by FcRs, at least in the NK cells tested *in vitro*. The potential influence of the *FCGR3A*F158V polymorphism, which affects FcRs binding to the Fc fragment of antibodies and is more prevalent among patients with MM and MGUS, remains to be investigated (27).

#### 2.2.1 Neonatal IgG Fc receptor

The Neonatal IgG Fc Receptor (FcRn) was characterized years ago from maternal milk. The roles of FcRn are distinct from those of receptors binding the Fc domains of different classes of immunoglobulins. Its primary functions identified so far are related to the metabolism of circulating IgG, including therapeutic monoclonal IgG, and albumin. These molecules are internalized into cytoplasmic compartments and then into endosomes. Similar internal processing occurs for antibodies, which are eventually extruded and released into biological fluids at neutral pH. This step is crucial for regulating the stability of therapeutic antibodies and directly determining their *in vivo* half-life (28).

FcRn also differs from other FcRs in terms of structure, regulation, and tissue distribution. FcRn are heterodimers composed of heavy and light molecular weight chains (29). The light chain consists of β2-microglobulin (β2m), a monomorphic component also shared with HLA Class I molecules. The heavy chain is encoded by the Fcϒ receptor and transporter (*FCGRT*) gene, located on chromosome 19q13.3. Although reminiscent of HLA-controlled products, suggesting potential yet unidentified functions, FcRn is not capable of presenting peptides. Its known ligands include IgG, albumin, and pan-echovirus. Upregulation of FcRn is primarily associated with stimulation via tumor necrosis factor (TNF), while other pathways have been identified in non-human models. Down-regulation is primarily achieved through JAK/STAT1 signaling mediated by interferon γ.

Among immune cells, FcRn is expressed by monocytes, macrophages, dendritic cells, and neutrophils, and is present at low levels in B lymphocytes but not detectable on T lymphocytes and NK cells. FcRn maintains the tissue distribution of IgG by virtue of its ability to mediate transcytosis, transporting IgG across polarized endothelial and some epithelial cells (30).

FcRn serves multiple active and passive roles in immunity. Its passive functions include recycling and transcytosis, which protect monomeric IgG from degradation and facilitate its transport across external cell membranes. Its active functions are more varied, linked to the involvement of IgG in triggering various immune responses. These physiological characteristics can be leveraged to enhance the persistence of IgG and albumin in the circulation.

To date, a comprehensive analysis of FcRn’s influence has not been conducted in the context of the anti-CD38 antibodies approved for *in vivo* use, whose Fc regions are not modified. Only Nguyen et al. have analyzed the *in vivo* dynamics of Isatuximab (31, 32).

### 2.3 CD38-controlled cellular activities and metabolic adaptation during MM

#### 2.3.1 CD38 as an enzyme

CD38 has been studied in MM therapy primarily due to its function as an ectoenzyme. Initially, the mechanisms of action of this enzyme were considered paradoxical because CD38 is predominantly localized on the cell surface, yet its final products are utilized within cytoplasmic compartments. Only recently has CD38 been described within the cytoplasm and internal organelles (5).

The amino acid sequence of the CD38 enzyme is highly conserved throughout evolution, indicating that its enzymatic function is likely its original role. Functions related to immunity and cell biology were probably acquired over its long evolutionary history (at least 750 million years), eventually leading to its duplication and the emergence of the *CD157* gene. *CD157* encodes a molecule that differs significantly in structure and tissue distribution, retaining only some of the enzymatic functions attributed to CD38 (33).

The primary substrate of CD38 is NAD^+^, a key molecule in energy production and signaling. NAD^+^ plays a crucial role in a broad network of metabolic enzymes, such as nicotinamide phosphoribosyltransferase (NAMPT), nicotinate phosphorybosiltransferase (NAPRT), Sirtuins, and Sterile α-Toll/interleukin receptor (TIR), as well as the recently identified TIR motif- containing 1 (SARM1) (3).

CD38 is overexpressed by malignant plasma cells, significantly affecting i) the enzymatic reactions of CD38 within the BM niche, ii) the types of CD38-associated ectonucleotidases (*e.g.*, CD203a, CD73, TRAP) involved in adenosine (ADO) production, and iii) the environmental metabolic conditions (*e.g.*, pH, oxygen levels) within the BM niche (33). While the role of CD38’s enzymatic activity in MM therapy resistance is hypothesized, it has not yet been confirmed (34).

#### 2.3.2 CD38 and extracellular NAD^+^ metabolism

Nucleotides have a second life as intercellular communicators and signal transducers in the BM niche (35).

NAD^+^ metabolism is governed by cell surface ectoenzymes, which work together to disassemble extracellular and intracellular nucleotides extruded from cells via active channeling mechanisms or by microvesicles (MVs). CD38 is the principal extracellular NAD^+^-consuming enzyme. In fact, *CD38* knockout mice exhibit significant increases in both extra- and intracellular NAD^+^ levels (36, 37).

Because of its ability to use NAD^+^ as a substrate, CD38 has been hypothesized to act as a metabolic sensor, limiting the duration of NAD^+^ half-life and signaling in the extracellular compartment. Besides serving as a substrate for CD38, NAD^+^ may also function as a cytokine, eliciting rapid functional responses mediated by specific purinergic type 2 (P2) receptors (34, 38).

The products of NAD^+^ disassembly mediated by extracellular CD38, namely ADPR and NAM, may undergo further metabolic transformations [(e.g., to adenosine monophosphate (AMP) or adenosine (ADO)], particularly when co-expressed with other ectonucleotidases in certain pathological environments, such as the bone marrow (BM) niche (13). This niche is a seemingly closed system where MM cells are in direct contact with other cellular components, all expressing a range of surface enzymes. The plasma assures contact among the different cells, allowing the storage and exchange of substrates and final reaction products. In addition to CD38, ectoenzymes present in the niche (whether surface or soluble molecules) include CD39 (ectonucleoside triphosphate diphosphohydrolase-1), CD73 (ecto-5′-nucleotidase), and CD203a (ectonucleotide pyrophosphatase/phosphodiesterase-1). CD39 and CD73 run the canonical pathway of ADO production starting from ATP. CD203a, also known as Plasma Cell-1 (PC-1), was the first surface marker identified on plasma cells and is an enzyme that also uses ATP as a substrate (39). Recent reports suggest that CD38 can use phosphorylated NAD^+^ (NADP^+^) as a substrate and, in the presence of CD203a and CD73 under low pH conditions, produce ADO as a final product (40). Lowering environmental pH is one of the strategies employed by MM cells to alter their metabolism and promote immune evasion. In line with this, the canonical pathway of ADO production (CD39/CD73) operates at a very low rate in these conditions (40).

A key question is whether the products of the CD38-dependent chain of ectoenzymes play a role in physiology and especially in pathology. The MM niche provides an ideal model to explore this. ADO levels in BM plasma samples from MM patients were compared with those from patients with precursor forms of monoclonal gammopathy of undetermined significance (MGUS) and smoldering MM (SMM). The catabolism of NAD^+^, ADPR, and AMP to ADO was higher in samples from patients with severe MM than in those with MGUS and SMM (41).

These experiments lead to several key conclusions. First, ADO is produced in the BM niches where MM cells grow; second, ADO predominantly derives from the non-canonical pathway mediated by CD38/CD203a/CD73; third, disease severity is directly correlated with ADO levels, which are higher in patients with more severe prognoses. These observations highlight the presence of metabolic modifications within the MM niche that may facilitate immune evasion strategies (41).

The high level of ADO detected in the BM niche may contribute to the overall anergic state observed in MM patients. Relevant to this review is the link between NAD^+^ levels and T cell-mediated responses against tumors, an area that has gained increasing attention with the recent development of pharmacological inhibitors targeting CD38’s enzymatic activities (42). Several small-molecule inhibitors, such as 78C, suppress the enzymatic activity of CD38, thereby reducing NAD^+^ consumption (42). However, these compounds have not yet been used clinically due to potential off-target effects. In contrast, inhibition of CD38 exoenzymatic activity using the Ab68 mAb reduced the production of ADPR (43). These findings regarding ADO production warrant further research.

Recent research by Cea et al. showed that upregulation of surface CD38 leads to intracellular NAD^+^ depletion, which induces mitochondrial alterations and increases oxidative stress (44). Conversely, the availability of different NAD^+^ precursors appears to improve NAD+ levels, acting as adjuvants to boost T lymphocyte responses (44).

CD38 primarily functions as a NAD+-glycohydrolase (42). Its main product, ADPR, can either be metabolized by the CD203a/CD73 exoenzymatic tandem into ADO (40), which binds to purinergic type 1 (P1) receptors (particularly A2A and A2B), or facilitate Ca2+ influx through TRPM2 channels (45). These processes modulate lymphocyte proliferation and T cell activation (42, 46).

#### 2.3.3 CD38 as an immune checkpoint in therapy

Based on this understanding, it is speculated that CD38 serves as an immune checkpoint regulating NAD^+^ and ADO homeostasis. Consequently, metabolic reprogramming of NAD^+^ regulation via CD38 inhibition has garnered attention as a potential strategy for immunotherapy. This hypothesis was validated in a preclinical melanoma model, where blocking CD38 expression in T lymphocytes boosted NAD^+^ levels, improving the effectiveness of adoptively transferred T cells (47). Targeting the ADO pathway may also increase the antitumor effects of drugs through various mechanisms, such as by boosting effector T and NK cell functions and inhibiting the immunosuppressive effects of myeloid-derived suppressor cells (MDSC) (48). Additionally, due to its participation in the CD38/CD203a/CD73 adenosinergic pathway, CD38 has been identified as an effective predictor of anti-PD-1 antibody-based checkpoint immunotherapy responses (49).

Beside reported pharmacological inhibitors (42), several new strategies aim to inhibit CD38 and increase NAD^+^ pool concentrations. These approaches include antibody-drug conjugates (ADCs), antibody recruiting molecules (ARMs), engineered toxin bodies (ETBs), bispecific T-cell engagers (BiTEs), and XmAb Fc domain technology. Preclinical studies and clinical trials evaluating CD38 inhibition are ongoing in patients with either solid tumors or hematological malignancies, including MM (3).

### 2.4 CD38 enzyme activity and therapeutic antibodies: potential influence on refractoriness

A crucial question is whether the binding of therapeutic CD38 antibodies interferes with the enzyme’s functional activity. The differing actions of Daratumumab and Isatuximab *in vivo* are summarized in recent reviews (50, 51), and their effects further analyzed in Horenstein A.L. et al. (2024, submitted).

The primary distinction between these antibodies lies in the epitopes of CD38 they target. Isatuximab interacts with a discontinuous epitope partially overlapping the catalytic site, whereas Daratumumab binds to two continuous amino acid sequences situated outside the enzymatic active site of CD38 (52, 53). These differences may reflect the methods by which the two antibodies were selected. Isatuximab was originally developed for its ability to operate via CDC, while Daratumumab was selected to enhance ADCC activity. Additionally, Isatuximab was chosen for its capacity to inhibit the ADP-ribosyl cyclase activity of CD38 (*i.e.*, *in vitro* formation of cADPR from NAD^+^) through an allosteric antagonism (49–51). Further comparative analysis has shown that Daratumumab reduces *in vitro* cADPR production catalyzed by CD38’s ADP-ribosyl cyclase activity, while Isatuximab exhibits a stronger inhibitory effect, reducing cyclase activity by approximately 70% (54, 55).

These findings indicate a partial allosteric inhibition of CD38’s enzymatic activity, disrupting the catalytic site of extracellular CD38 responsible for *in vitro* formation of cADPR. However, the validity of these experimental conditions is limited by two factors, namely: i) CD38 produces multiple enzymatic products (56), and ii) antibody binding does not fully represent the enzymatic machinery. Notably, the inhibition of CD38’s extracellular cyclase activity accounts for only 2% of the molecule’s total enzymatic activity when targeted by therapeutic antibodies (4).

An alternative view suggests that the *in vitro* conditions used in these studies might not adequately represent *in vivo* dynamics. Nevertheless, the overarching conclusion is that while Daratumumab moderately affects CD38’s *in vitro* cyclase activity, Isatuximab exerts a stronger effect. Interestingly, both antibodies mildly activate CD38’s hydrolase activity (*i.e.*, the formation of ADPR from cADPR as a substrate) (57, 58). Importantly, it remains unclear whether the *in vitro* reduction of cADPR levels produced by extracellular CD38 in the presence of therapeutic mAbs leads to reduced intracellular Ca^2+^ mobilization and decreased signaling potential. On the other hand, potentially increased ADPR levels could contribute to immune suppression through ADO. Further investigations are essential to understand these complex interactions, especially regarding *in vivo* NAD^+^ homeostasis and tumor survival in closed systems. Other potential strategies to address therapeutic resistance are summarized in (59).

#### 2.4.1 Other potential methods for overcoming anti CD38 mAbs resistance: the role of CD47

CD47, an integrin-associated receptor, is widely expressed on the surface of many cell types, including red blood cells and cancer cells (60). Within the immunoglobulin family, it functions as a “don’t eat me” signal. Its ligands include thrombospondin-1 (TSP-1), signal-regulatory protein α (SIRPα), and integrins (61). When CD47 binds to SIRPα on dendritic cells and macrophages, it activates an immunoreceptor tyrosine-based inhibitory motif that suppresses phagocytosis (61). Blocking this signaling axis enhances the elimination of tumor cells by macrophages and neutrophils (62). CD47 is highly expressed in solid tumors and hematologic malignancies, and this overexpression correlates with poor prognosis in various cancers (60, 63). In the context of MM cancer cells upregulate CD47 to evade immune surveillance, exploiting this pathway to enhance their survival.

CD47 expression increases as the disease progresses from MGUS to MM (64). Numerous studies have shown promising preclinical results for anti-CD47 therapies in treating hematologic malignancies. These therapies are effective either as standalone treatments or in combination with clinically approved drugs, including those for MM (63, 65–67). Anti-CD47 therapy alone has shown efficacy against MM (64, 68), and its therapeutic impact is improved when combined with CD38-targeting agents. Storti et al. reported that treatment with daratumumab increases MM cell death, especially in the presence of a subset of CD14^+^/CD16^+^ monocytes, and that combining daratumumab with anti-CD47 enhances the elimination of MM cells that are resistant to daratumumab alone (66).

Bispecific antibodies (BsAbs) are also emerging as promising tools in cancer therapy. These antibodies exhibit higher affinity and binding activity for CD38 than for CD47, reducing potential on-target and off-tumor effects. A study by Li et al. explored various CD38/CD47 BsAbs, each with unique *in vitro* properties: IMM5605–26B4 displayed the most potent inhibitory effect on CD38 enzymatic activity, while IMM5605–12C10 was effective in directly killing MM cells and completely eradicating established tumors in mouse models (69). ISB 1442, a human bispecific antibody was recently reported. The antibody has one arm equipped with biparatopic sequences reacting with distinct epitopes of the CD38 molecule. The second arm is designed to react at high affinity with CD47. The basic strategy is to obtain a reagent focusing its activity prevalently around the cells expressing CD38, which enables avidity-induced blocking of CD47 on the same cells, in this way avoiding unwanted off-tumor effects. The reagent was also equipped with a modified domain with enhanced FcR functions. ISB 1442 showed enhanced tumor killing *in vitro* compared to daratumumab on myeloma cells with varying CD38 expression levels. IBS 1442 is currently in a Phase I clinical trial for relapsed refractory multiple myeloma (NCT05427812) (70).

### 2.5 Next generation anti-CD38 antibodies

Advances in antibody therapy may also derive from the development of new anti-CD38 reagents that retain specificity but feature significant modifications in their structure and functions. Some modifications concern the antibody’s ability to react with the family of FcRs, to avoid mechanical trapping in tissues or the implementation of FcR-mediated signals interfering with the therapeutic process.

Of the numerous anti-CD38 reagents currently undergoing evaluation, summarized below are representative examples of antibodies now being designed for therapy.

1. Pre-clinical evaluation of the monoclonal antibody CM313. This conventional antibody was developed by selecting a precise CD38 sequence and identifying unique complementary-determining regions, which provided much higher sensitivity and improved performance compared to traditional reagents. The original murine antibody was humanized, and CM313 has been shown to inhibit some of the enzymatic activities of CD38. Toxicology studies in *Cynomolgus* monkeys and murine models indicate that CM313 is well tolerated (71).
2. The Danish company GenMab introduced specific mutations in the Fc domain of a human anti-CD38 antibody, leading to (or improving) hexamer formation upon binding to cell surface CD38. The strategy adopted is reported in a recent paper (72). This approach increased CDC function in MM cells (even in cells expressing low levels of surface CD38) and was also effective against other hematological malignancies. The hexabody did not compete *in vitro* with Daratumumab or Isatuximab, but strongly inhibited the cyclase activity of the target. Furthermore, the hexabody induced FcR-mediated signals in NK cells and macrophages, while other effectors involved in the immune response were apparently unaffected. Clinical trials evaluating the safety and efficacy of the reagent *in vivo* are ongoing (NCT04824794) (73).
3. Bispecific antibodies feature two distinct Fabs which can lead to the generation of effects similar to those evoked by CAR-T cells (which are beyond the scope of this review). Of the two arms, one is designed to provide tumor specificity, while the other has the role of triggering the signaling potential. This last step elicits an anti-tumor response by T lymphocytes or by NK cells. The use of bispecific reagents has found wide clinical applications because of its simplicity and–equally important–lower costs (74).
4. A further strategy was to increase the function of the T lymphocytes, by activating two distinct activation pathways. Sanofi (Europe and USA) designed SAR442257, a trispecific antibody where CD38 confers target specificity and CD3 and CD28 provide dual activation signals to optimize T lymphocyte effector functions and to ensure sustained proliferation. An original characteristic of this reagent is the choice of CD28-mediated signals to help prevent cell death, which is often observed after T-cell activation. The trispecific reagent was evaluated for its ability to induce activation and proliferation of T lymphocytes. Cytokine release syndrome (CRS) was avoided by using an IgG4 isotype, which does not activate complement fixation (67).
  CD28 is not only expressed by T lymphocyte but also by myeloma cells (75). Preclinical tests have shown that 86% of RRMM patients express CD28, compared to newly diagnosed MM patients. The conclusion is that the trispecific SAR442257 operates via dual targeting of CD38 and CD28 on myeloma cells and of CD3 and CD28 on T lymphocytes, showing superior MM killing compared to bispecific antibodies. The study also highlighted the role of TNF-β in favoring resistance to the antibody. The TGF-β inhibitor Vactoserib enhances sensitivity of the target cells to the antibody (76).
  The same antibody was analyzed *ex vivo* using Myeloma Drug Sensitivity Testing to identify suitable MM patients for this therapy. To this end, tests were conducted by challenging the patients’ endogenous T lymphocytes with primary MM cells. This novel approach enabled the identification of patients who responded to SAR442257. The association among T-cell phenotype, performance, disease state and sensitivity to this antibody is currently under investigation (77). The trispecific antibody was also recently tested on acute myeloid leukemia cell lines and patients (78).
5. An alternative strategy was to flank CD3 engager with co-targeting CD38 and BCMA. The ISB 2001 antibody is a recombinant trispecific complex: one arm contains a sequence reacting with CD38, while the second arm contains specificities for CD3 and BCMA. The results obtained by an international group are apparently superior to other trispecific antibodies, as shown *in vivo* in mouse models. The group has also devised a quantitative systems pharmacology model, which exploits clinical patient data to obtain a personalized posology (79).
6. Another strategy to improve overall binding efficiency involves biparatopic antibodies. These recombinant reagents use two heavy chains that react with distinct epitopes of the same molecule. The California-based company Teneobio targeted CD38 with the aim of developing an antibody capable of fully inhibiting the enzymatic activities of the molecule–not yet achieved with the anti-CD38 reagents currently used in *in vivo* therapy. The platform used for constructing these antibodies included two Fabs that react with two distinct sequences of the CD38 domain hosting the catalytic site. Additionally, the antibody was improved with a silenced IgG4 Fc, which does not deplete or interfere with the normal effectors (80). The TNB-T38 antibody is now in trials. The reagent has been instrumental in demonstrating a relationship between the products generated by the CD38 enzyme and multi-organ fibrosis (43).
7. Another original approach was adopted by Candelaria et al., who switched the class of Ig, using IgE instead of the conventional IgG. The potentially beneficial features of IgE include its affinity for the FcϵR, expressed by the immune effectors responsible for ADCC and ADCP, and the low levels of normal IgE in the blood, which avoids competition with the therapeutic IgE. *In vitro* results are promising, showing induction of degranulation in FcϵR-expressing cells. Increased survival was observed in mice when disseminated tumor cells were exposed to the IgE antibody and PBMC as a source of monocyte effectors (81).

### 2.6 Areas to be investigated to expand research in the field of CD38

#### 2.6.1 Fate of unbound therapeutic antibody

One proposal is the selection of therapeutic antibodies that show negligible reactivity with red blood cells (RBC) and platelets, which are reported to express CD38 at a cell surface density too low to trigger lysis. Despite their reduced ability to capture the therapeutic antibodies, the high number of these cells suggests that they could act as carriers of anti-CD38 antibodies in biological fluids. Thus, RBC and likely platelets in biological fluids may sequester a portion of the antibody pool away from their therapeutic targets (82).

#### 2.6.2 Tumor microenvironment

S. Paget’s “seed and soil theory” from 1889 predicted the role of TME in tumor growth. This concept has been supported by numerous studies across various types of cancer (83). The core concept is that normal components of the tissue (or of the niche where the tumor grows) may provide, either occasionally or as part of a precise neoplastic strategy, a set of soluble factors that contribute to, or even determine, the growth of the tumor itself or facilitate immune evasion.

The TME consists of multiple elements: in addition to epithelial, stromal, and immune cells, recent interest has been focused on cancer-associated fibroblasts (CAF) and adipocytes.

##### 2.6.2.1 Cancer-associated fibroblasts (CAFs)

Traditionally considered as tumor-supporting, recent reports suggest they also play a role in inhibiting tumor development. This dual role may be attributable to the heterogeneity and plasticity of cancer-associated fibroblastsCAFs), largely dependent on their origin. Newly proposed classifications may help translate their functional characteristics into therapeutic strategies (84, 85). This therapeutic potential has been confirmed in lung (85) and breast cancers (86). The connection between MM and CAFs is increasingly recognized, as outlined in the following recent publications (87–91):

##### 2.6.2.2 Adipocytes

Recently acknowledged as potential contributors to myeloma dynamics and immune cell functions, adipocytes are abundant in the BM and increase with age–a relevant factor given MM’s prevalence in older individuals. Unlike traditional classifications of brown, beige, and white fat, BM adipocytes exhibit distinct characteristics and functions (92, 93). Single-nucleus RNA sequencing (snRNA-seq) has identified a subpopulation of BM adipocytes involved in thermogenesis (94). Notably, forward-feedback loops between adipocytes and malignant plasma cells in the BM niche can modulate MM cell proliferation and support MM growth. Some of these interactions involve the upregulation of CD38, mediated by adipocyte-secreted angiotensin II (Ang II), which has been identified as the direct cause of increased expression of acetyl-CoA synthetase (ACSS2) in MM plasma cells, promoting growth by stabilizing the oncoprotein IRF4 *in vitro* and *in vivo* (95).

CD38 can also influence sirtuin activities, affecting histone acetylation and gene expression. This suggests a potential link between ACSS2 and CD38 in regulating gene expression through epigenetic mechanisms.

Moreover, extracellular CD38 is involved in the conversion of NAD^+^ to adenosine (ADO), leading to NAD^+^ depletion (40). ADO plays a role in regulating intercellular crosstalk as well as thermogenic and metabolic functions, such as adipocyte differentiation and lipid synthesis, in adipose tissues (96).

Recent evidence highlights the active and complex roles of BM adipocytes, suggesting that they are not merely passive energy stores but actively regulate multiple functions that can influence hematopoiesis, for instance, either positively or negatively (97).

Additionally, multiple reports confirm that adipocytes secrete a range of paracrine factors relevant to MM dynamics, notably stem cell factor, adiponectin, leptin, and IL-2β. MM plasma cells have also been shown to modulate inflammatory cytokine production, which can influence adipocyte function and lipolysis. For a comprehensive review, see reference (98).

Further, adipocytes are also studied in relation to myeloma progression. An analysis of an MGUS sample revealed a significant upregulation of adipogenic commitment. Moreover, adipocytes are reported to modulate responses to treatment. Specifically, Ochiai M et al. (99) demonstrated that adipocytes mediate drug resistance in MM plasma cells, contributing to recurrence and/or disease progression (99).

**Figure 2:**
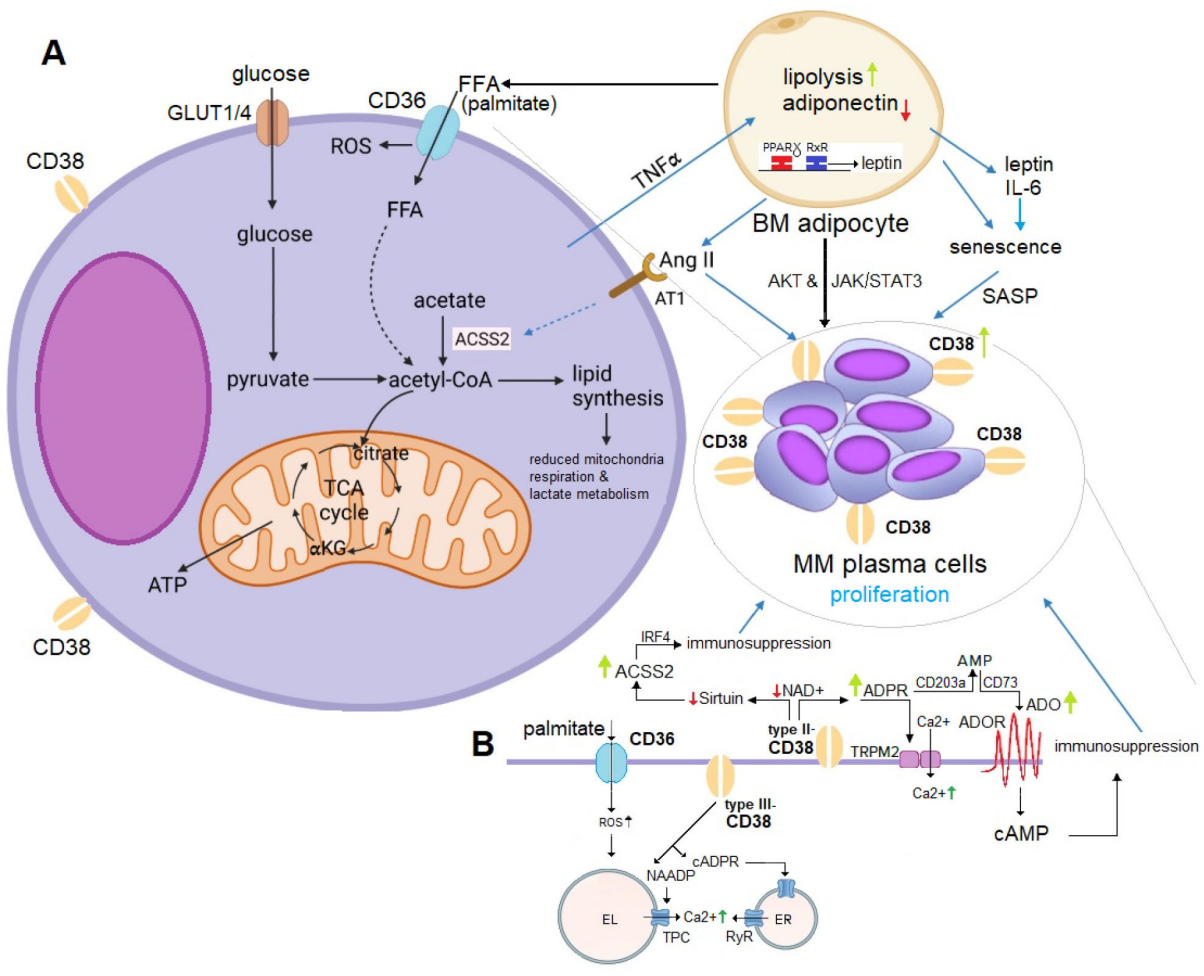
**(A)** Mechanistic Role of Adipocyte-Derived ACSS2 in MM Proliferation. Adipocyte-derived ACSS2 generates acetyl-CoA, fueling the TCA cycle and driving fatty acid (FFA) synthesis (e.g., palmitate). This promotes MM proliferation through multiple mechanisms: (1) stabilizing the oncogenic protein IRF4 via sirtuin-mediated histone acetylation, and (2) supporting lipogenesis, which induces metabolic changes favoring MM cell survival. Adiponectin inhibits MM growth but is downregulated by malignant plasma cells via TNFα signaling. Conversely, leptin and IL-6 stimulate proliferation through the AKT and JAK/STAT3 pathways. IL-6 also induces a SASP phenotype in BM adipocytes, leading to upregulation of CD38 expression in MM plasma cells. Additionally, lipolysis in BM adipocytes releases FFA, which MM plasma cells uptake to further enhance their proliferation. Elevated Ang II levels from BM adipocytes activate CD38 overexpression, promoting ADPR-to-ADO conversion (detailed in panel **B**), and contributing to immunosuppression and IRF4-mediated MM growth. **(B)** CD36-CD38 Crosstalk in Lipid and NAD^+^ Metabolism. ACSS2-derived palmitate induces ER stress and lipotoxicity by activating NF-κB, producing ROS, and activating the NLRP3 inflammasome. The uptake of palmitate via CD36 influences substrate availability for NAD+ synthesis, affecting CD38 activity and disrupting metabolic homeostasis. CD36 and CD38 expression are regulated by PPARγ, which forms a heterodimer with RXRα to control genes involved in FFA uptake, adipocyte differentiation, and inflammation. PPARγ activation upregulates CD38, increasing NAADP production (via type III-CD38) and intracellular Ca2+ levels to support MM cell survival. CD38-overexpressing MM cells deplete NAD^+^, impairing sirtuin activity and disrupting histone acetylation and gene expression. This interaction emphasizes the interplay between lipid metabolism and epigenetic regulation in MM proliferation. ACSS2, acetyl-CoA synthetase 2; ADO, adenosine; Ang II, angiotensin II; AT1, angiotensin II receptor type 1; EL, endolysosomes; ER, endoplasmic reticulum; FFA, free fatty acids; GLUT1/4, glucose transporter 1/4; PPARγ, proliferator-activated receptor-γ; RXRα, retinoic X receptor-α; ROS, reactive oxygen species; SASP; senescence-associated secretory phenotype; TCA cycle, tricarboxylic acid cycle; TNFα, tumor necrosis factor alpha.

The literature indicates that MM may stimulate lipolysis in adipocytes through the activation of specific pathways. The resulting free fatty acids (FFA) are then taken up by MM cells via fatty acid transporter proteins, which support their proliferation within the BM niche (100–102). **Figure 2** provides a schematic illustration of the homeostatic feedback circuits between adipocytes and MM cells.

The link between obesity and MM remains largely correlative. However, research in this area is rapidly expanding, including studies that investigate the role of senescence. Beyond scientific interest, these findings have translational relevance, as modulating adipocyte function or addressing eating behaviors could serve as potential targets for adjunctive therapies in MM (95, 101). A recent review by C. Marques-Mourlet et al. provides a comprehensive overview of the mechanistic contributions to MM, summarized in a clear model [see **Figure 2**, reference (103)]. Although these observations are primarily based on murine studies (104), they suggest a promising path toward rapid confirmation and potential translation into clinical settings.

## 3 Conclusions

CD38 antibody therapy has marked a significant advancement in managing myeloma, providing a valuable proof-of-concept for exploring *in vivo* questions derived from basic science. The positive clinical outcomes were accompanied by unforeseen effects that had not been predicted by conventional *in vitro* immunology. These complexities are further underscored by a recent report by Soriani et al., which suggests that CD38 can act as a decoy for extracellular cGAMP, a mediator of innate immunity. The relevance of these observations is currently being validated in *in vivo* models (105).

One critical challenge in clinical settings has been the emergence of antibody refractoriness in MM patients. This resistance was initially thought to be secondary to a significant decrease in surface CD38 following antibody exposure. Here, basic science has provided insights into the molecular regulation of CD38, as well as a link between CD38 expression, retinoids, and other emerging drug combinations.

A deeper understanding of how the antibody delivers its lethal hits or channels signals is also key. Antibody action on cell targets is mediated by FcRs, which then activate CDC, ADCC, ADCP, and direct signaling. This system, however, is more complex than initially thought. For instance, complement activation is regulated by inhibitory receptors, adding another layer of complexity to the process.

The benefits observed in antibody therapy derive from the multiple effects induced by IMiDs, including a synergistic enhancement of surface CD38 expression and the simultaneous inhibition of complement inhibitory receptors. Additional inhibitory actions are exerted by IMiDs on suppressor regulatory cells, and their influence may also vary according to the antibody used (50, 51).

However, observations from *in vivo* studies complicate the picture even further. For example, patients refractory to IMiDs in earlier lines of therapy regained sensitivity to lenalidomide or pomalidomide following *in vivo* treatment with Daratumumab (106). This evidence suggests a multifaceted scenario, where the antibody not only targets and eliminates tumor cells through specific mechanisms but also simultaneously activates various pathways in killer cells while inhibiting and depleting suppressor cells.

Further evidence supporting CD38’s role as a signaling molecule comes from studies targeting CD38 with soluble agonists, derived from traditional Chinese medicine, as was the case for the family of retinoids. These promising results are still awaiting confirmation in human *in vivo* models (107).

A unifying hypothesis may come from the notion that the effects driven by an antibody result from a combination of target binding via the Fab domains and simultaneous interaction of the Fc domain with specific receptors. The mechanisms underlying this heterotypic cross-talk are not yet fully understood, nor are the various effects observed in normal and pathologic conditions (*e.g.*, inflammation, autoimmunity and–relevant to this review–responsiveness to antibody therapy) (108).

When applying these observations to the MM model, which lacks FcR, the lytic effects must be mediated by cells expressing FcR. The action of the therapeutic antibody on effectors, which often display both the CD38 target and FcRs, is harder to dissect. At high *in vivo* concentrations, the therapeutic antibody may shift the balance toward these effector cells. As a result, the effectors are influenced by signals from the ligation of the target molecule by the Fab-binding site of the antibody and the simultaneous engagement of the Fc domain of the IgG by the different FcRs.

**Figure 3:**
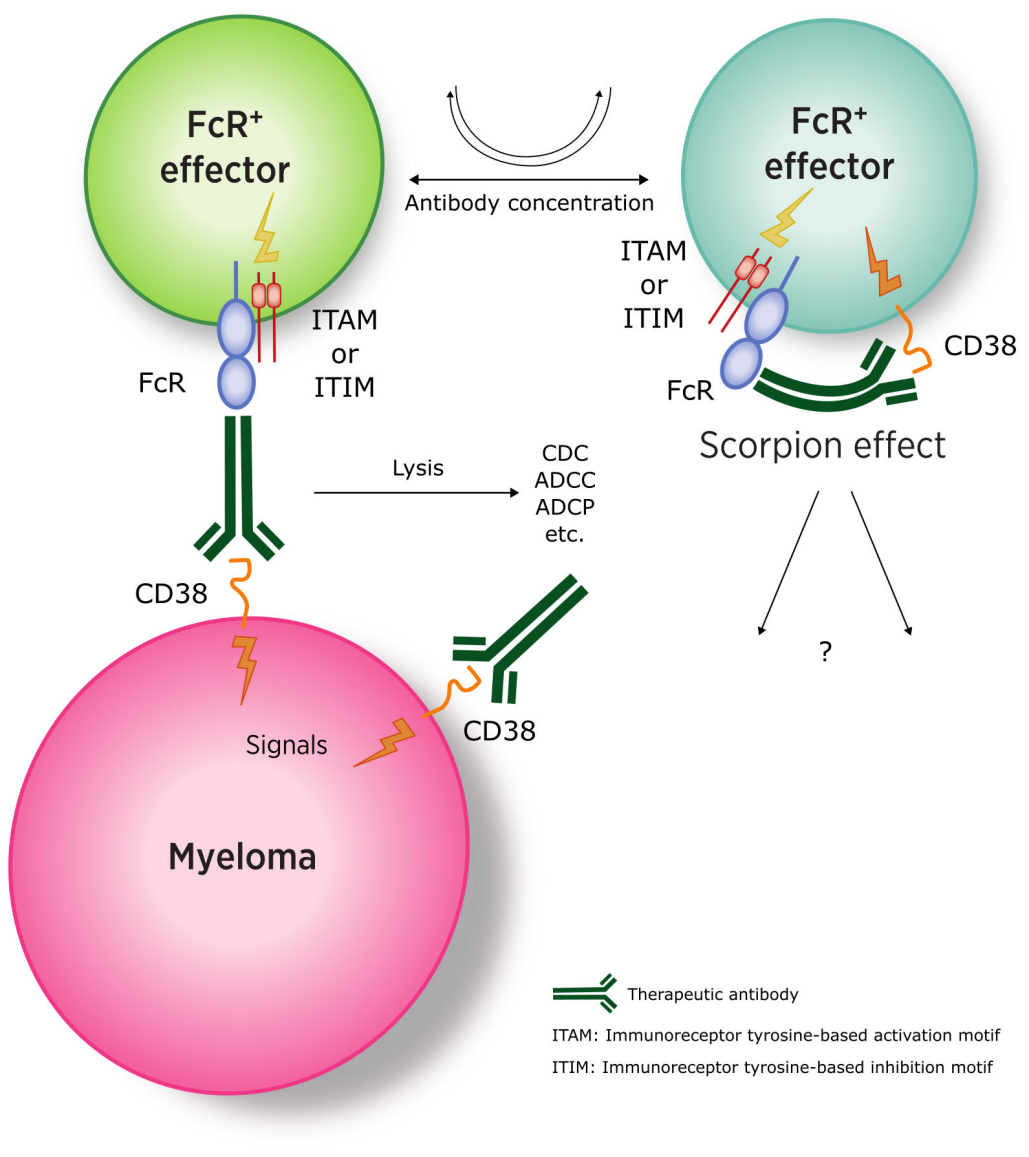
Interaction between anti-CD38 antibodies and myeloma (FcR-negative) and effector cells (CD38-and FcR-positive). (ITAM, immunoreceptor tyrosine-based activation motif; ITIM, immunoreceptor tyrosine-based inhibitory motif). Adapted from (109).

The balance between activating and inhibitory receptors ultimately determines the therapeutic outcome. While the reaction of the therapeutic antibody seems relatively straightforward on the myeloma side (as MM cells are FcR-negative), more complex interactions occur with effector cells (FcR^+^), where the antibody can engage both the Fab-binding site and one of the FcRs on the same cell (**Figure 3**) (110).

In conclusion, understanding these complex interactions is key to advancing myeloma therapy and for achieving significant improvements in patient outcomes. Although a definitive cure has yet to be found, the treatment landscape has dramatically changed over the past decade, with monoclonal antibodies playing a central role in this progress.
